# Supplementary material for: Content-rich biological network constructed by mining PubMed abstracts
Source: BMC Bioinformatics. 2004 Oct 8;5:147. doi: 10.1186/1471-2105-5-147 (PMC528731; doi:10.1186/1471-2105-5-147)
Supplement: Additional File 5 — The original Chilibot query results of the term "long-term potentiation (LTP)" and 22 other terms, limiting the latest references analyzed to the years 1990, 1995, 2000, and 2004. [file 1471-2105-5-147-S5.bz2 › chilibotAdditionalFile5/ltp2000/html/TAU_SYNAPTOTAGMIN.html]

 


 **TAU** and **SYNAPTOTAGMIN** 
  
Found 3 abstracts in PubMed,  **3 abstracts were retrieved and analyzed**.  


---

 Search Google  |
 PDF files only 
|  EDU domain only 

---

**Interactive relationship** (e.g. stimulation, inhibition, etc)

**Parallel relationship** (e.g. studied together, co-existance, homology, etc.)

- In this study, we have found that, during the first 10 postnatal days 1 10 PN, the facial motor nucleus lacked immunoreactivity for synaptophysin,  **synaptotagmin** , GAP 43,  **tau** , and SNAP 25.  Ref: 8725306 J Comp Neurol, 1996
- In contrast, the hypoglossal motor nucleus displayed immunoreactivity from 1 PN for synaptophysin,  **synaptotagmin** , SNAP 25, GAP 43,  **tau** , and MAP 2.  Ref: 8725306 J Comp Neurol, 1996
- After 10 PN, immunoreactivity increased in the facial motor nucleus for synaptophysin,  **synaptotagmin** , GAP 43, and  **tau** .  Ref: 8725306 J Comp Neurol, 1996
